# Accuracy and Acceptability of Wrist-Wearable Activity-Tracking Devices: Systematic Review of the Literature

**DOI:** 10.2196/30791

**Published:** 2022-01-21

**Authors:** Federico Germini, Noella Noronha, Victoria Borg Debono, Binu Abraham Philip, Drashti Pete, Tamara Navarro, Arun Keepanasseril, Sameer Parpia, Kerstin de Wit, Alfonso Iorio

**Affiliations:** 1 Department of Health Research Methods, Evidence, and Impact McMaster University Hamilton, ON Canada; 2 Department of Medicine McMaster University Hamilton, ON Canada; 3 School of Interdisciplinary Sciences McMaster University Hamilton, ON Canada; 4 Department of Oncology McMaster University Hamilton, ON Canada; 5 Department of Emergency Medicine Queen's University Kingston, ON Canada

**Keywords:** diagnosis, measurement, wrist-wearable devices, mobile phone

## Abstract

**Background:**

Numerous wrist-wearable devices to measure physical activity are currently available, but there is a need to unify the evidence on how they compare in terms of acceptability and accuracy.

**Objective:**

The aim of this study is to perform a systematic review of the literature to assess the accuracy and acceptability (willingness to use the device for the task it is designed to support) of wrist-wearable activity trackers.

**Methods:**

We searched MEDLINE, Embase, the Cochrane Central Register of Controlled Trials, and SPORTDiscus for studies measuring physical activity in the general population using wrist-wearable activity trackers. We screened articles for inclusion and, for the included studies, reported data on the studies’ setting and population, outcome measured, and risk of bias.

**Results:**

A total of 65 articles were included in our review. Accuracy was assessed for 14 different outcomes, which can be classified in the following categories: count of specific activities (including step counts), time spent being active, intensity of physical activity (including energy expenditure), heart rate, distance, and speed. Substantial clinical heterogeneity did not allow us to perform a meta-analysis of the results. The outcomes assessed most frequently were step counts, heart rate, and energy expenditure. For step counts, the Fitbit Charge (or the Fitbit Charge HR) had a mean absolute percentage error (MAPE) <25% across 20 studies. For heart rate, the Apple Watch had a MAPE <10% in 2 studies. For energy expenditure, the MAPE was >30% for all the brands, showing poor accuracy across devices. Acceptability was most frequently measured through data availability and wearing time. Data availability was ≥75% for the Fitbit Charge HR, Fitbit Flex 2, and Garmin Vivofit. The wearing time was 89% for both the GENEActiv and Nike FuelBand.

**Conclusions:**

The Fitbit Charge and Fitbit Charge HR were consistently shown to have a good accuracy for step counts and the Apple Watch for measuring heart rate. None of the tested devices proved to be accurate in measuring energy expenditure. Efforts should be made to reduce the heterogeneity among studies.

## Introduction

### Background

Tracking, measuring, and documenting one’s physical activity can be a way of monitoring and encouraging oneself to participate in daily physical activity; increased activity is thought to translate into important positive health outcomes, both physical and mental [1]. In the past, most physical activity tracking was done manually by oneself or an external assessor, through records, logbooks, or using questionnaires. These are indirect methods to quantify physical activity, meaning that they do not measure movement directly as it occurs [2]. The main disadvantages of such methods are the administrative burden on either the self-assessor or the external assessor and the potential imprecision because of recall bias [2,3]. Direct methods to assess physical activity [2], such as accelerometers or pedometers that digitally record movement, are preferred because they eliminate recall bias and are convenient. This process of activity tracking has become automated, accessible, and digitized with wearable tracking technology such as wristband sensors and smartwatches that can be linked to computer apps on other devices such as smartphones, tablets, and PCs. When data are uploaded to these devices, users can review their physical activity log and potentially use this feedback to make behavior changes with regard to physical activity.

The ideal device should be acceptable to the end user, affordable, easy to use, and accurate in measuring physical activity. Accuracy can be defined as the closeness of the measured value to the actual value. Accuracy can be calculated using measures of agreement, sensitivity and specificity, receiver operating characteristic curves, or absolute and percentage differences [4]. Agreement can be defined as “the degree of concordance between two or more sets of measurements” [5]. It can be measured as percentage agreement, that is, the percentage of cases in which 2 methods of measurements of the same variable lead to classification in the same category. Another example of methods of calculating agreement is the κ statistic, which measures agreement beyond chance [6]. Sensitivity and specificity are the true positive and true negative proportions, respectively. These proportions are calculated using the measurement method that we are evaluating as the index test and another method, known to be accurate, as the reference standard [4]. Receiver operating characteristic curves are obtained plotting the sensitivity versus the complement of specificity and can be used to find optimal cutoff points for the index test. Absolute and percentage differences are used to determine how far the index test measurement is from the reference standard or their average [4]. Acceptability can be widely defined as “the demonstrable willingness within a user group to employ information technology for the task it is designed to support” [7]. It can be assessed qualitatively (eg, through questionnaires or interviews) or quantitatively (eg, percentage of the time during which the device is worn or the data are available or measured using ad hoc scales). On the basis of a 2019 review, acceptability or acceptance of wrist-wearable activity-tracking devices is dependent on the type of user and context of use [8]. This same review indicates that research on accuracy has not kept up with the plethora of wearable physical activity–tracking devices in the market [8]. This may be because of the rapidly changing landscape as companies continue to upgrade models with different technical specifications and features. The purpose of this systematic review is to assess the acceptability and accuracy of these wrist-wearable activity-tracking devices through a focused in-depth review of primary studies assessing these 2 characteristics.

### Objectives

The first objective of this systematic review is to assess the accuracy of wrist-wearable activity-tracking devices for measuring physical activity.

The second objective is to assess the acceptability of wrist-wearable activity-tracking devices for measuring physical activity.

## Methods

The methods used for this systematic review have been registered in the PROSPERO database (CRD42019137420).

### Search Strategy

The databases searched were MEDLINE, Embase, the Cochrane Central Register of Controlled Trials, and SPORTDiscus from inception to May 28, 2019. Search strategies were developed to retrieve content on wearable activity trackers and on their accuracy and reproducibility of results. We used search terms, including *Wearable device* and *Fitness tracker*, to identity studies on the use of a consumer-based wearable activity tracker, whereas terms such as *data accuracy* and *reproducibility of results* were included to bring in content focused on activity tracker validation. The search strategy is available on the web in the PROSPERO record. A snowball search was conducted by checking the references of relevant studies and systematic reviews on this topic that were identified in our original search.

### Selection of Studies

For the acceptability objective, the population was the general population, without sex or age restrictions. The intervention was the use of a wrist-wearable activity tracker. The outcome was any quantitative measure of acceptability, including wearing time, data availability, and questionnaires to assess acceptability.

For the accuracy objective, the population was again the general population, the index test had to be a wrist-wearable activity tracker, and the reference standard could be another device or any method used to measure physical activity, including questionnaires and direct observation. The outcome could be any measure of physical activity, including but not limited to step count, heart rate, distance, speed, activity count, activity time, and intensity of physical activity.

For both objectives, this review examined both research-grade devices (activity trackers available only for research purposes) and commercial devices (those available to the general public). The included studies were limited to the community-based everyday-life setting. Laboratory tests such as research studies were included as long as everyday settings were reproduced, thereby excluding patients who were institutionalized and those who were hospitalized. We set no restrictions on the length of observation for the original studies.

The exclusion criteria included the following: device not worn on the wrist, studies measuring sleep, and studies on patients who were institutionalized or hospitalized.

All studies reporting primary data were considered for inclusion, with the exception of case reports and case series.

Using the aforementioned inclusion and exclusion criteria and a piloted form, we initially screened for inclusion from the titles and abstracts of the retrieved articles, using the web-based software Rayyan (Rayyan Systems Inc) [9]. Subsequently, we screened the full texts of the studies identified as potentially eligible from the title and abstract screening for selection.

### Data Extraction and Risk-of-Bias Assessment

Data were extracted to an Excel (Microsoft Corp) file. The data extraction form was based on a previous publication on the same topic [8] and adapted to the needs of this review. The following data were extracted: general study information: first author’s name, publication year, type of study (prospective vs retrospective and observational vs interventional), duration of follow-up (in days), and setting (laboratory vs field); characteristics of the population: number of participants, underlying health condition (eg, healthy participants, people with severe obesity, and chronic joint pain), gender, and age distribution (mean and SD or median and minimum–maximum or first and third quartiles); measures of accuracy: step count, distance, speed, heart rate, activity count, time spent being active, and intensity of physical activity; and acceptability of the device, including but not limited to data availability, wearing time, ease of use. The risk of bias was assessed using the Quality Assessment of Diagnostic Accuracy Studies, version 2, tool [10]. This tool guides the assessment of the risk of bias in diagnostic accuracy studies in 4 domains: patient selection, index test, reference standard, and flow and timing. We rated the risk of bias in each domain as *High*, *Probably high*, *Probably low*, and *Low*. When necessary, the study authors were contacted for additional information.

Throughout title and abstract and full-text screening and the data extraction, each step was performed in duplicate with 2 reviewers (NN and BAP) deciding independently on inclusion or exclusion and, if needed, later having a discussion with another author to make a final decision. Disagreements were solved through discussion and, when needed, with the intervention of a third reviewer (FG, VBD, or DP). The reviewers were trained with calibration exercises until an adequate performance was achieved for each of these steps.

### Diagnostic Accuracy Measures

When available, we extracted the mean absolute percentage error (MAPE) or the mean percentage error. When these were not available, we extracted other measures in the following order of priority: mean difference, mean bias (Bland–Altman), accuracy determined through intraclass correlation coefficient, and correlation coefficient (Pearson or Spearman). When the outcome was dichotomized and sensitivity and specificity were calculated, we reported on these values. When available, we reported measures of variability or 95% CIs for all the aforementioned measures. The formulas used for calculating the MAPE, mean percentage error, mean difference, and mean bias are reported in Multimedia Appendix 1 [11-75]*.*

### Synthesis of Results

Because of the significant heterogeneity observed in the studies’ populations, settings, devices assessed, reference standards, outcomes assessed, and the outcome measures reported, we decided not to perform a quantitative synthesis and have provided a narrative synthesis of the results for both the objectives. For the accuracy objective, given the high number of studies retrieved, we summarized results only for devices that were included in at least two studies reporting the same outcome. All the remaining results are reported in Multimedia Appendix 1.

### Ethics Approval

This systematic review was based on published data and therefore did not require a submission to a research ethics board.

### Availability of Data and Materials

Most of the data that support the findings of this study are available in Multimedia Appendix 1. A guide on how to use the database provided in Multimedia Appendix 1 can be found in Multimedia Appendix 2. The full data set can be made available upon reasonable request.

### Code Availability

This is not applicable to this systematic review because no quantitative data synthesis was performed.

## Results

### Overview

**Figure 1:**
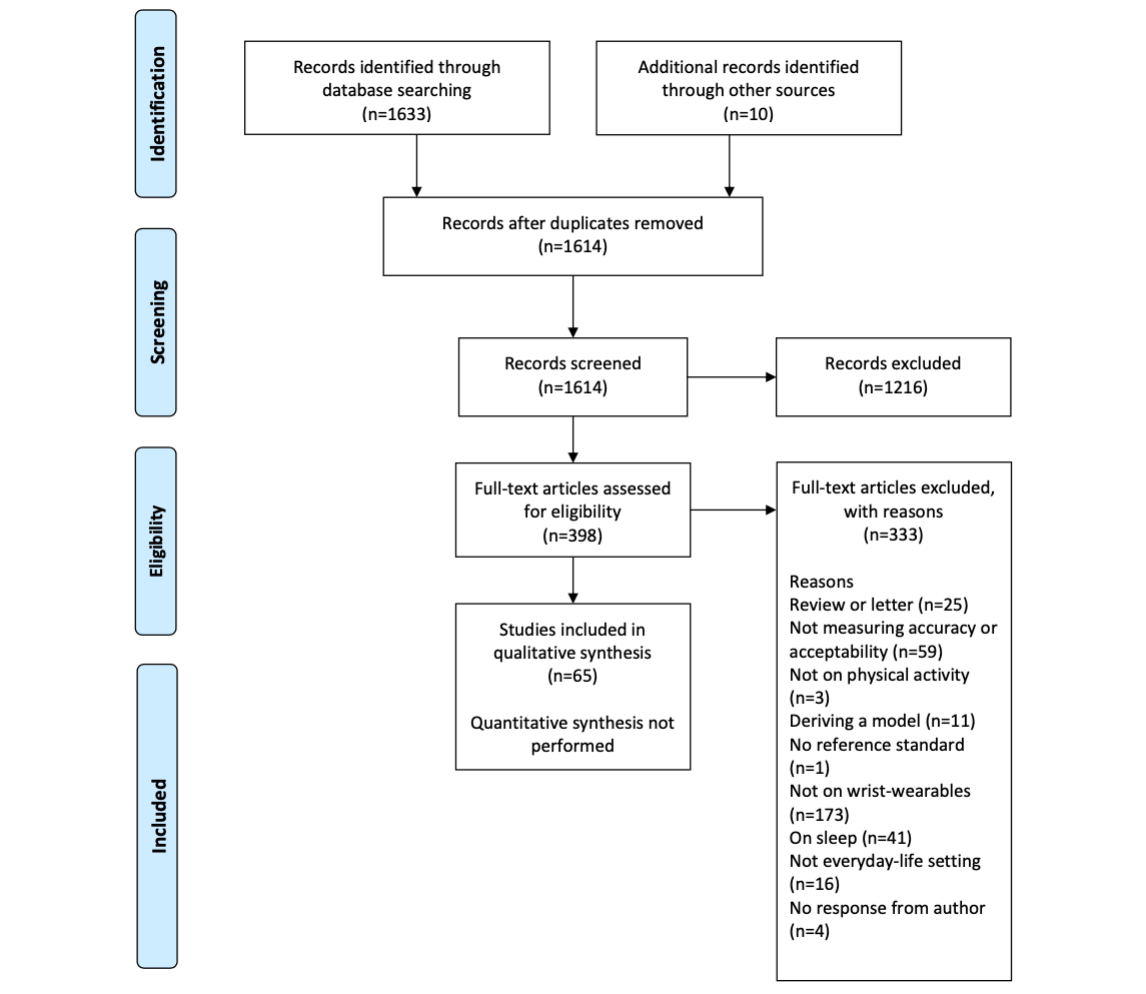
PRISMA (Preferred Reporting Items for Systematic Reviews and Meta-Analyses) 2009 study flow diagram.

The search identified 1633 records (1614, 98.84%, after the removal of duplicates). The study flow diagram is presented in Figure 1. After screening the full texts of 398 articles, 65 (16.3%) were included in the systematic review. The characteristics of the included studies are summarized in Table 1 and Multimedia Appendix 3 [11-67] for the accuracy objective and Table 2 for the acceptability objective. All the included studies were single-center studies, with a prospective, observational design. The complete results for accuracy and acceptability have been reported in Multimedia Appendix 1.

**Table 1:** Characteristics of the studies reporting on accuracy (N=57).

First author, year	Setting	FUP^a^ time, days	Sample, n	Age (years), mean (SD)	Female, %	Underlying health condition	Outcome	Device brand and model
Alharbi [11], 2016	Laboratory	<1	48	66 (7)	48	Patients undergoing cardiac rehabilitation	Step count and MVPA^b^	Fitbit Flex
Alsubheen [12], 2016	Field	5	13	40 (12)	38	Healthy	Step count and energy expenditure	Garmin Vivofit
An [13], 2017	Laboratory and field	1	35	31 (12)	51	Healthy	Step count	Fitbit Flex, Garmin Vivofit, Polar Loop, Basis B1 Band, Misfit Shine, Jawbone UP24, and Nike FuelBand SE
An [14], 2017	Field	<1	62	24 (5)	40	Healthy	Active time	ActiGraph GT3X
Blondeel [15], 2018	Field	14	8	65 (8)	25	COPD^c^	Step count	Fitbit Alta
Boeselt [16], 2016	Field	3	20	66 (7)	15	COPD	Step count, energy expenditure, and MVPA	Polar A300
Bruder [17], 2018	Laboratory and field	395	32	—^d^	—	Rehabilitation after radial fracture	Activity count	ActivPAL
Bulathsinghala [18], 2014	Laboratory	1	20	70 (10)	—	COPD	Physical activity intensity	ActiGraph GT3X+
Burton [19], 2018	Laboratory	<1	31	74 (6)	65	Healthy older adults	Step count	Fitbit Flex and Fitbit Charge HR
Choi [20], 2010	Laboratory	<1	76	13 (2)	62	Healthy	Energy expenditure	ActiGraph GT1M
Chow [21], 2017	Laboratory	1.5	31	24 (5)	39	Healthy	Step count	ActiGraph wGT3xBT-BT, Fitbit Flex, Fitbit Charge HR, and Jawbone UP24
Chowdhury [22], 2017	Laboratory and field	2	30	27 (6)	50	Healthy	Energy expenditure	Microsoft Band; Apple Watch, series not specified; Jawbone Up24; and Fitbit Charge
Cohen [23], 2010	Laboratory and field	3	57	70 (10)	—	COPD	Speed	ActiGraph Mini Motionlogger
Compagnat [24], 2018	Laboratory	<1	46	65 (13)	—	Stroke	Energy expenditure	ActiGraph GT3X+
Dondzila [25], 2018	Laboratory and field	3-62	40	22 (2)	58	Healthy	Step count, energy expenditure, and heart rate	Fitbit Charge HR and Mio Fuse
Dooley [26], 2017	Laboratory	1	62	23 (4)	58	Healthy	Heart rate and energy expenditure	Apple Watch, series not specified; Fitbit Charge HR; and Garmin Forerunner 225
Durkalec-Michalski [27], 2013	Laboratory	2	20	26 (5)	55	Healthy	Energy expenditure	ActiGraph GT1M
Falgoust [28], 2018	Laboratory	1	30	—	—	Healthy	Step count	Fitbit Charge HR, Fitbit Surge, and Garmin Vivoactive HR
Ferguson [29], 2015	Field	2	21	33 (10)	52	Healthy	Step count, MVPA, and energy expenditure	Nike FuelBand, Misfit Shine, and Jawbone UP
Gaz [30], 2018	Laboratory and field	<1	32	36 (8)	69	Healthy	Step count and distance	Fitbit Charge HR; Apple Watch, series not specified; Garmin Vivofit 2; and Jawbone UP2
Gillinov [31], 2017	Laboratory	<1	50	38 (12)	54	Healthy	Heart rate	Garmin Forerunner 235; TomTom Spark; Apple Watch, series not specified; and Fitbit Blaze
Gironda [32], 2007	Laboratory	<1	3	43^e^	31	Pain syndromes	Activity count	Actiwatch Score
Hargens [33], 2017	Laboratory and field	7	21	31^e^	68	Healthy	MVPA, energy expenditure, and step count	Fitbit Charge
Hernandez-Vicente [34], 2016	Field	7	18	21 (1)	50	Healthy	Energy expenditure, vigorous active time, active time, and step count	Polar V800
Huang [35], 2016	Laboratory	1	40	24 (3)	25	Healthy	Step count and distance	Jawbone UP24, Garmin Vivofit, Fitbit Flex, and Nike FuelBand
Imboden [36], 2018	Laboratory	<1	30	49 (19)	50	Healthy	Energy expenditure, MVPA, and step count	Fitbit Flex, Jawbone Up24, and Fitbit Flex
Jo [37], 2016	Laboratory	<1	24	25^e^	50	Healthy	Heart rate	Basis Peak K and Fitbit Charge
Jones [38], 2018	Laboratory	118	30	33^e^	—	Healthy	Step count	Fitbit Flex
Kaewkannate [39], 2016	Laboratory	<1	7	31 (0)	14	Healthy	Step count	Fitbit Flex, Jawbone UP24, Withings Pulse, and Misfit Shine
Lamont [40], 2018	Laboratory	<1	33	67 (8)	64	Parkinson disease	Step count	Garmin Vivosmart HR and Fitbit Charge HR
Lauritzen [41], 2013	Laboratory and field	<1	18	—	56	Older adults	Step count	Fitbit Ultra
Lawinger [42], 2015	Laboratory	<1	30	26 (6)	70	Healthy	Activity count	ActiGraph GT3X+
Lemmens [43], 2018	Laboratory	<1	40	31 (5)	100	Parkinson disease	Energy expenditure	Philips optical heart rate monitor
Magistro [44], 2018	Laboratory	<1	40	74 (7)	60	Healthy	Step count	ADAMO Care Watch
Mandigout [45], 2017	Laboratory	<1	24	68 (14)	60	Stroke	Energy expenditure	Actical and ActiGraph GTX
Manning [46], 2016	Laboratory	<1	9	15 (1)	—	Severe obesity	Step count	Fitbit One, Fitbit Flex, Fitbit Zip, ActiGraph GT3x+, and Jawbone UP
Montoye [47], 2017	Laboratory	<1	30	24 (1)	47	Healthy	Step count, energy expenditure, and heart rate	Fitbit Charge HR
Powierza [48], 2017	Field	<1	22	22 (2)	55	Healthy	Heart rate	Fitbit Charge
Price [49], 2017	Laboratory	<1	14	23^e^	21	Healthy	Energy expenditure	Fitbit One, Garmin Vivofit, and Jawbone UP
Redenius [50], 2019	Laboratory	4	65	42 (12)	72	Healthy	MVPA	Fitbit Flex
Reid [51], 2017	Field	4	22	21 (2)	100	Healthy	MVPA and step count	Fitbit Flex
Roos [52], 2017	Laboratory	2	20	24 (2)	40	Runners	Energy expenditure	Suunto Ambit, Garmin Forerunner 920XT, and Polar V800
Schaffer [53], 2017	Laboratory	<1	24	54 (13)	42	Stroke	Step count	Garmin Vivofit
Scott [54], 2017	Field	7	89	—	54	Healthy	Daily mean activity and MVPA	GENEActiv
Semanik [55], 2020	Laboratory	7	35	52^e^	69	Chronic joint pain	MVPA	Fitbit Flex
Sirard [56], 2017	Laboratory and field	7	14	9 (2)	50	Healthy	Energy expenditure, MVPA, and step count	Movband and Sqord
St-Laurent [57], 2018	Laboratory	7	16	33 (4)	100	Pregnant	Step count and MVPA	Fitbit Flex
Stackpool [58], 2013	Laboratory	<1	20	22 (1)	50	Healthy	Step count and energy expenditure	Jawbone UP, Nike FuelBand, Fitbit Ultra, and Adidas miCoach
Stiles [59], 2013	Laboratory	1	10^e^	39 (6)	100	Healthy premenopausal women	Loading rate (BW^f^/s)	GENEActiv and ActiGraph GT3X+
Støve [60], 2019	Laboratory	<1	29	29 (9)	41	Healthy	Heart rate	Garmin Forerunner
Tam [61], 2018	Laboratory	<1	30	32 (9)	50	Healthy	Step count	Fitbit Charge HR and Xiaomi Mi Band 2
Thomson [62], 2019	Laboratory	<1	30	24 (3)	50	Healthy	Heart rate	Apple Watch, series not specified; and Fitbit Charge HR2
Wahl [63], 2017	Laboratory	<1	20	25 (3)	50	Healthy	Step count, energy expenditure, and distance	Polar Loop, Beurer AS80, Fitbit Charge HR, Fitbit Charge, Bodymedia Sensewear, Garmin Vivofit, Garmin Vivosmart, Garmin Vivoactive, Garmin Forerunner 920XT, Xiaomi Mi Band, and Withings Pulse
Wallen [64], 2016	Laboratory	<1	22	24 (6)	50	Healthy	Heart rate, energy expenditure, and step count	Apple Watch, series not specified; Samsung Gear S; Mio Alpha; and Fitbit Charge
Wang [65], 2017	Laboratory	<1	9	22 (1)	44	Healthy	Step count	Huawei B1, Xiaomi Mi Band, Fitbit Charge, Polar Loop, Garmin Vivofit 2, Misfit Shine, and Jawbone UP
Woodman, 2017 [66]	Laboratory	<1	28	25 (4)	29	Healthy	Energy expenditure	Garmin Vivofit, Withings Pulse, and Basis Peak
Zhang [67], 2012	Laboratory	1	60	49 (7)	62	Healthy	Activity classification (sedentary, household, walking, and running)	GENEActiv

^a^FUP: follow-up.

^b^MVPA: moderate- to vigorous-intensity physical activity.

^c^COPD: chronic obstructive pulmonary disease.

^d^Not available.

^e^SD not reported.

^f^BW: body weight.

**Table 2:** Characteristics of the studies reporting on acceptability (N=11).

First author, year	Setting	FUP^a^ time, days	Sample, n	Age (years), mean (SD)	Female, %	Underlying health condition	Outcome assessed	Device brand and model
Boeselt [16], 2016	Laboratory and field	7	20	66 (7)	15	COPD^b^	Ease of use and other characteristics	Polar A300
Deka [68], 2018	Field	5	46	65 (12)	67	CHF^c^	Data availability	Fitbit Charge HR
Farina [69], 2019	Field	2	26; 26	80 (6); 76 (6)	39; 73	Dementia; caregivers of patients with dementia	Wearing time	GENEActiv
Fisher [70], 2016	Field	7	34	69^d^	—^e^	Parkinson disease	Ease of use and other characteristics	AX3 data logger
Kaewkannate [39], 2016	Field	<1	7	31 (0)	14	Healthy	Ease of use and other characteristics	Fitbit Flex, Jawbone UP24, Withings Pulse, and Misfit Shine
Lahti [71], 2017	Laboratory	120	40	—	—	Schizophrenia	Data availability	Garmin Vivofit
Marcoux [72], 2019	Field	46	20	73 (7)	20	Idiopathic pulmonary fibrosis	Data availability	Fitbit Flex 2
Naslund [73], 2015	Field	80-133	5	48 (9)	90	Serious mental illness	Wearing time	Nike FuelBand
Speier [74], 2018	Laboratory	90	186	—	—	Coronary artery disease	Wearing time	Fitbit Charge HR2
St-Laurent [57], 2018	Laboratory	1	16	33 (4)	100	Pregnant	Ease of use and other characteristics	Fitbit Flex
Rowlands [75], 2018	Field	425	1724	13 (1)	100	Healthy	Data availability	GENEActiv

^a^FUP: follow-up.

^b^COPD: chronic obstructive pulmonary disease.

^c^CHF: congestive heart failure.

^d^SD not reported.

^e^Not available.

### Accuracy

**Figure 2:**
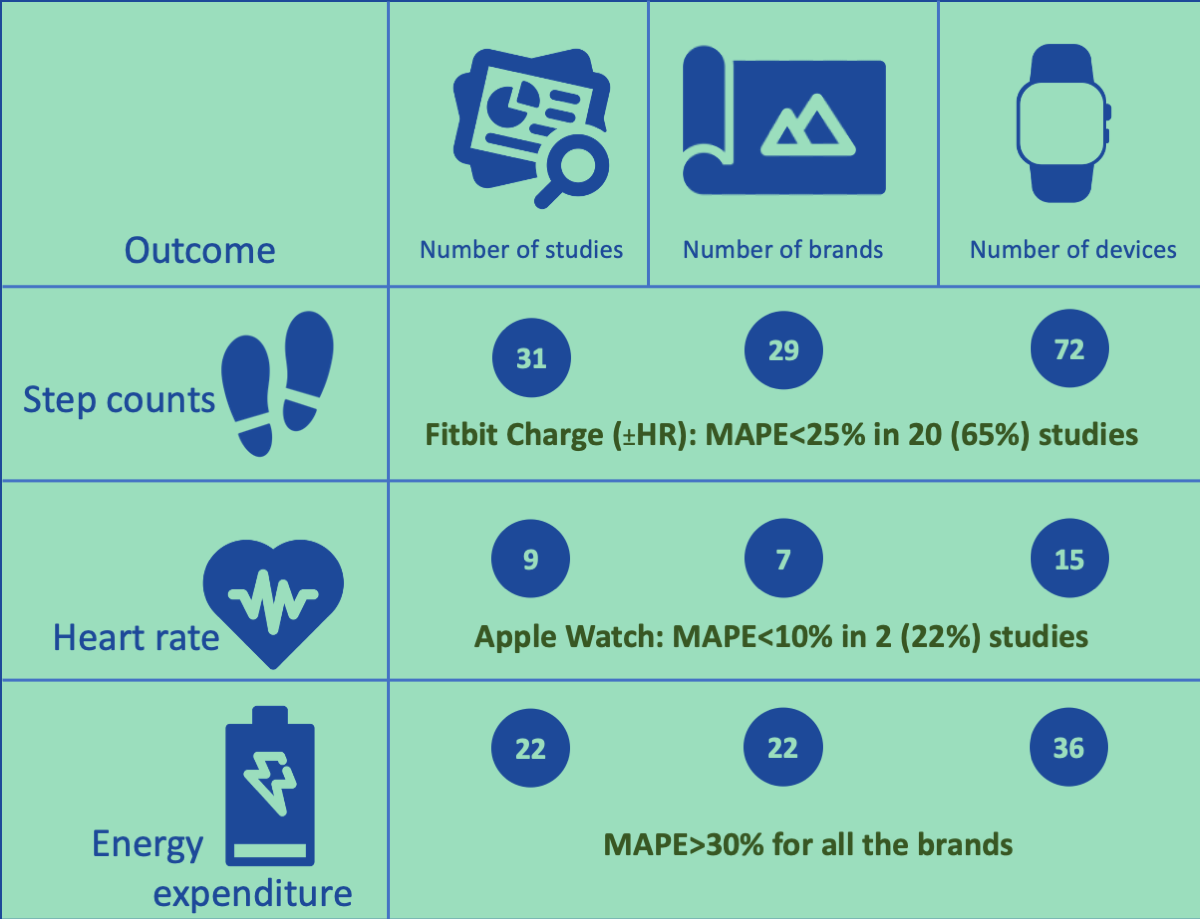
Summary of the results for the main accuracy outcomes. MAPE: mean absolute percentage error.

The accuracy of wrist-wearable activity trackers was assessed in 57 studies on 72 devices from 29 brands. Step count, heart rate, and energy expenditure (EE) were the most commonly assessed outcomes in the appraised literature. The results of these outcomes are summarized in Figure 2 (icons by Nikhil Bapna, Yoyon Pujiyono, Chintuza, Gregor Cresnar, Andrejs Kirma, and Yigit Pinarbasi from the Noun Project [76]), in which we have highlighted the standout device for the most frequently reported outcomes.

### Step Counts

A total of 31 studies on 72 devices from 29 brands reported data on step counts. The reference standards used were manual count (directly observed or on video, usually with the help of a tally counter) or automated count through video analysis, an activity tracker (8 different devices), or a photoelectric cell.

The *ActiGraph wGT3xBT-BT*, tested against manual count, showed a mean percentage error of –41.7% (SD 13.5%) [21]. The *ActiGraph GT3x+* showed no statistically significant correlation with the same reference standard [46].

The *Apple Watch* (series not specified) was evaluated in 6% (2/31) of studies using manual count as the reference standard [30,64]. The mean difference between the device and the manual count varied from –47 (SD 470) steps to 39.44 (SD 151.81) steps in different walking conditions.

For the *Fitbit Alta,* the mean step count was 773 (SD 829) higher (*P*=.009) than the one obtained with the reference standard, an accelerometer [15]. For the *Fitbit Charge*, the mean difference was –59 (SD 704) steps compared with direct observation [64]. The MAPE for the same device ranged from –4.4% to 20.7%, using different automated step count methods as the reference standard [33,63,65]. The *Fitbit Charge HR* was assessed in 29% (9/31) of studies, using direct observation [19,21,28,30,61] or an automated method of step count as the reference standard [25,40,47,63]. The MAPE ranged from –12.7% to 24.1%. The accuracy of the *Fitbit Flex* in measuring steps was assessed in 35% (11/31) of studies, using manual count [13,19,21,35,36,38,39,46] or an ActiGraph device [11,51,57] as the reference standard. The mean percentage error ranged from –23% to 13%. For the *Fitbit One* and *Fitbit Zip*, no statistically significant correlation was found in step counting using direct observation as the reference standard [46]. The correlation coefficient was not reported. For the *Fitbit Surge***,** the mean difference compared with direct observation was –86.0 steps (*P=*.004) [28]. For the *Fitbit Ultra*, the MAPE was 99.6% (SD 0.8%) [41] and the Pearson correlation coefficient against manual count ranged from 0.44 to 0.99 in different exercise conditions [58].

The accuracy of the *Garmin Vivofit* was assessed in 16% (5/31) of studies [12,13,35,53,63], with a MAPE ranging from –41% to 18% [13,53,63]. For the *Vivofit 2***,** a study reported a MAPE of 4% [65] and another study reported a mean difference ranging from 5.09 (SD 8.38) steps to 98.06 (SD 137.49) steps in different walking conditions (over a maximum distance of 1.6 km) [30].

In a study by Wahl et al [63], the MAPE against automated step counting using a photoelectric cell as the reference standard, in different exercise types and conditions, ranged from –2.7% to 1.5% for the *Garmin Forerunner 920XT*, from –1.5% to 0.6% for the *Garmin Vivoactiv*, and from –1.1% to –0.3% for the *Garmin Vivosmart* [63]. For the *Garmin Vivoactive HR*, the mean difference against manual step count was –19.7 steps (*P=*.03) [28]. For the *Garmin Vivosmart HR*, the mean difference ranged from –39.7 (SD 54.9) steps to 5.4 (SD 5.8) steps for different walking speeds and locations (outdoor vs indoor) over a total of 111-686 steps [40].

For the *Jawbone UP*, the MAPE was –6.73% in a study [65] and the mean absolute difference 806 over an average of 9959 steps in another study [29]. For the *Jawbone UP2*, the mean difference ranged from 16.19 (SD 29.14) steps to 64 (SD 66.32) steps for different walking conditions over a maximum distance of 1.6 km [30]. For the *Jawbone UP24*, the mean percentage error ranged from –28% to –0.8% [21,35,36].

For the *Misfit Shine*, the MAPE ranged from –13% to 23% [13,65].

For the *Mio Fuse*, the MAPE ranged from –5% to –16% at different treadmill speeds [25], whereas in another study, the mean percentage error was <5% for the *Xiaomi Mi Band 2* [61].

For the *Nike FuelBand***,** the mean percentage error ranged from –34.3% (SD 26.8%) to –16.7% (SD 16.5%) [35], whereas for the *FuelBand SE*, the MAPE ranged from 10.2% to 45.0% [13].

The MAPE for the *Polar Loop* ranged from –13% to 27% in 3 studies [13,63,65]. Regarding 2 other devices from *Polar*, for the *A300*, a Pearson correlation coefficient of 0.96 (*P*<.01) [16] was reported, whereas for the *V800*, the Bland–Altman bias was equal to 2487 (SD 2293) steps per day over a mean 10,832 (SD 4578) steps per day measured with the reference standard [34].

For the *Withings Pulse*, the MAPE for step count ranged from –16.0% to –0.4% [63] and the accuracy from 97.2% to 99.9% [39]. All the remaining devices were only used in 1 study each, and the results are reported in Multimedia Appendix 1*.*

### Heart Rate

A total of 9 studies on 15 devices from 7 brands evaluated the accuracy of activity-tracking devices to measure the participants’ heart rates. The reference standards used were electrocardiography, pulse oximetry, or another activity tracker (4 different devices).

For the Apple Watch, the MAPE for measuring heart rate ranged from 1% (SD ~1%) to 7 (SD ~11%) [26,31].

In the *Fitbit* family of devices, for the *Fitbit Charge*, the mean bias estimated with the Bland–Altman method ranged from –6 (SD 10) bpm to –9 (SD 8) bpm [37,48,64]. For the *Fitbit Charge HR* or *Fitbit Charge* *HR2*, the MAPE for measuring heart rate ranged from 2.4% (SD ~1.5%) to 17% (SD ~20%) [26,47,62]. For the *Fitbit Blaze*, the MAPE ranged from 6% (SD 6%) to 16% (SD 18%) for different activities [31].

### Active Time: Time Spent in Moderate- to Vigorous-Intensity Physical Activity and Other Outcomes

A total of 13 studies on 11 devices from 8 brands reported on the time spent being active, most frequently defined as the time spent in moderate- to vigorous-intensity physical activity (MVPA; 11 studies), expressed in minutes per day. The reference standard for MVPA was another activity tracker (3 different devices). Other outcomes were time spent being active (standing+walking+running), time spent running, or time spent on different types of physical activity, with each of these outcomes being reported in only 1 study.

For the *Fitbit Flex*, the MAPE for measuring the time spent in MVPA varied from 7% (SD 6%) to 74% (SD 13%) [50] and the mean percentage error ranged from –65% to 10% [11,36]. All the other devices were only used in 1 study each, and the results are reported in Multimedia Appendix 1.

### Intensity of Activity: EE and Other Outcomes

A total of 24 studies on 42 devices from 23 brands focused on measuring the intensity of physical activity. The most frequent measure of intensity was EE, expressed as kcal, evaluated in 92% (22/24) of studies. The less frequent measures of intensity included loading rate and the classification of physical activity (sedentary, household, walking, and running). For EE, the reference standard used most commonly was indirect calorimetry (6 different instruments). Less common reference standards included EE estimated with other wearable activity trackers (5 different devices), estimated based on the treadmill settings, or direct room calorimetry.

Among the *ActiGraph* family, the mean percentage difference in the EE compared with the reference standard in people with previous stroke was 3% for walking participants and 47% for participants with wheelchair using the *ActiGraph GT3X+* [24]. The Spearman correlation coefficient was 0.08 (*P=*.71) if worn on the plegic side and 0.20 (*P=*.34) if worn on the nonplegic side with the *ActiGraph GTX* [45]. Using the *ActiGraph GT1M*, the mean percentage difference was 0.5% (SD 8.0%) in a study [20], whereas another study found that the device overestimated EE at moderate intensity by 60% and underestimated EE by 40% at vigorous intensity while being 86% accurate in measuring EE at light intensity [27].

For the *Apple Watch***,** the MAPE for EE ranged from 15% (SD 10%) to 211% (SD ~96%) [22,26].

In the *Fitbit* family, the MAPE from the *Charge* model ranged from –4.5% to 75.0% in different studies [22,33,63] and from –12% to 89% for the *Charge HR* [25,26,47,63]. For the *Fitbit Flex,* a mean percentage bias of –13% was reported [36]. For the *Fitbit One,* a study reported a mean bias of 2.91 (SD 4.35) kcal per minute [49], whereas for the *Fitbit Ultra,* the Pearson correlation coefficient ranged from 0.24 to 0.67 for different physical activities [58].

Among the devices from *Garmin***,** the MAPE for EE ranged from –21% to 45% for the *Vivofit* [63,66], from –2% to –36% for the *Vivosmart* [63], and from 5% to 37% for the *Vivoactive* [63].

For the *Garmin Forerunner*, the MAPE ranged from –27% to 49% for the model *920XT* [52,63] and from 31% (SD ~26%) to 155% (SD ~164%) for the model 225 [26].

In the *Polar* family, the MAPE for EE ranged from 10% to 40% for the *V800* model [52], with a Bland–Altman bias of 957.5 (SD 679.9) kcal, when the mean EE measured with the reference standard was 1456.48 (SD 731.40) kcal [34]. For the *Polar Loop*, the MAPE for EE ranged from 6% to 56% [63]. The Pearson correlation coefficient was 0.74 (*P*<.01) for the *Polar A300* [16].

For the *Withings Pulse*, the MAPE for EE ranged from –39% to 64% [63,66].

### Outcomes Reported Less Frequently

Other outcomes that were evaluated less frequently include distance, reported in 3 studies on 15 devices from 7 brands, always using the measured distance as the reference standard [30,35,63]; speed, reported in a study using 1 device, with actual speed (on a treadmill) as the reference standard [23]; and activity count, defined as the number of activities (eg, number of arm movements or body movements based on observation or measured acceleration data), reported from 4 studies on 4 devices from 4 different brands using as the reference standard manual count (video recording), video analysis (automated), or an activity tracker [17,32,42,54].

### Risk of Bias

The risk-of-bias assessment for each outcome is reported in Multimedia Appendix 1. In summary, all the studies were at high or probably high risk of bias for the domain *Patient selection* because they used a convenience sampling technique. Almost all the studies were at low risk of bias for the domains *Index test* and *Reference standard* because the 2 measurement methods were applied at the same time and interpreted without knowledge of the results obtained with the other method. A small number of studies was identified as high risk for the domain *Flow and timing* based on the high percentage (>25%) of missing data for the index test or reference standard.

### Acceptability

The acceptability of wrist-wearable activity trackers was assessed in 11 studies on 10 devices from 9 brands.

### Data Availability

In all, 36% (4/11) of studies focused on data availability, expressed as a proportion of time in which the data were available, and a different device was used in each of these studies. The denominator for the proportion could be the study duration or the time spent exercising. Rowlands et al [75] found that data availability was 52% in a pediatric healthy population using the GENE*Activ* for 14 months. Deka et al [68] focused on data availability during exercise time. In this study, adult patients with cardiac heart failure activated their *Fitbit Charge HR* in 75% of the exercise sessions (over 5 days) and data were available for 99% of the time when activated. Marcoux et al [72] studied the *Fitbit Flex 2* in adults with idiopathic pulmonary fibrosis (for 46 days). Of the 20 patients, 2 did not succeed in activating the device. Among the remaining participants, data were available for a mean of 91% (SD 20%) of the time. Lahti et al [71] studied the *Garmin Vivofit* in adults with schizophrenia and found data available for 97% of the time (over 4 months).

### Wearing Time

In all, 27% (3/11) of studies reported on the wearing time. Farina et al [69], using the *GENEActiv*, found that 89% of the participants with dementia and 86% of their caregivers wore the device for the duration of the study (28 days). Speier et al [74], using the *Fitbit Charge 2*, enrolled participants with coronary artery disease. The median time spent wearing the activity tracker ranged from 44% to 90% over 90 days. Finally, for *Nike FuelBand***,** in a study on patients with schizophrenia, the mean wearing time was 89% (SD 13%) over 80-133 days [73].

### Ease of Use and Other Characteristics

In all, 36% (4/11) of studies focused on the ease of use and similar characteristics of wrist-wearing devices. The *Polar A300* was assessed in patients with chronic obstructive pulmonary disease wearing the device for 3 days using the Post-Study System Usability Questionnaire, which calculates a score that ranges from 1 to 7 (the lower the better) for 3 subdomains [16]. The mean scores were 1.46 (SD 0.23) for system quality, 2.41 (SD 0.53) for information quality, and 3.35 (SD 0.62) for interface quality. The *AX3 data logger* was assessed in persons with Parkinson disease wearing the device for 7 days [70]. A questionnaire created ad hoc was used for the assessment; 94% of the participants agreed that they were willing to wear the sensors at home, and 85% agreed that they were willing to wear the sensors in public. However, some of the participants reported problems with the strap fitting and the material (number not reported). The *Fitbit Flex* was assessed with a questionnaire created ad hoc in a study on pregnant women followed for 7 days [57]. The *Fitbit Flex* was reported by 31% to be inconvenient, 6% to be poorly esthetic, and 12% to be uncomfortable. Kaewkannate et al [39] asked healthy participants to wear 4 different devices over 28 days and compared them using a questionnaire created ad hoc. The *Withings Pulse* had the highest satisfaction score, followed by *Misfit Shine, Jawbone UP24,* and *Fitbit Flex.*

## Discussion

### Study Findings

We systematically reviewed the available evidence on the acceptability and accuracy of wrist-wearable activity-tracking devices for measuring physical activity across different devices and measures. We found substantial heterogeneity among the included studies. The main sources of heterogeneity were the studies’ population and setting, the device used, the reference standard, the outcome assessed, and the outcome measure reported.

Acceptability was evaluated in 11 studies on 10 devices from 9 brands. Data availability was ≥75% for the Fitbit Charge HR, Fitbit Flex 2, and Garmin Vivofit. Data availability is defined as the amount of data captured over a certain time period, which, in this case, is over a predetermined duration of each respective study. Data availability can be a measure of how accurate a device is at capturing data when the device is worn. For example, if an individual wears the device for 8 hours but only 4 hours of data are available, some questions may be raised on the capability of the device to capture information accurately. The wearing time was 89% for both the GENEActiv and Nike FuelBand. Wearing time is defined as the amount of time the device is worn over a predetermined duration for each study. For each study, wearing time may have been assessed differently; for example, a study may measure wearing time over a day, whereas another study may measure over a week. Both data availability and wearing time can provide a deeper look into acceptability because participants may wear a device more frequently and, ultimately, have more data available if a device is more acceptable. Accuracy was assessed in 57 studies on 72 devices from 29 brands. Among 14 outcomes assessed, step counts, heart rate, and EE were the ones used most frequently. For step counts, the Fitbit Charge (or the Fitbit Charge HR) had a MAPE <25% across 20 studies. For heart rate, the Apple Watch had a MAPE <10% in 2 studies. For EE, the MAPE was >30% for all the brands, showing poor accuracy across devices.

### Comparison With Other Systematic Reviews

Feehan et al [77] conducted a systematic review on the accuracy of Fitbit devices for measuring physical activity. The review did not specifically focus on wrist-wearable activity trackers; it also included studies using activity trackers worn on other body locations (torso, ankle, or hip). This systematic review reported a good accuracy of Fitbit devices in measuring steps, with 46% of the included studies reporting a measurement error within –3% to +3%. Regarding EE, the authors concluded that “Fitbit devices are unlikely to provide accurate measures of energy expenditure.” Studies on heart rate were not included in the review. Evenson et al [78] performed a systematic review focusing on Fitbit and Jawbone devices. Similarly, wearing the device on the wrist was not an inclusion criterion. The authors concluded that for step counts, the included studies often showed a high correlation, with the correlation coefficient ≥0.80 among devices from both brands, with the reference standards. The correlation was frequently low for the outcome EE. Similar to the review by Feehan et al [77], the outcome heart rate was not included in this systematic review. The results of these systematic reviews are consistent with our findings for the devices and outcomes assessed.

### Strengths and Limitations

The main strengths of our systematic review include the inclusion of all the devices reported in the literature; the reporting on all the outcomes related to acceptability and accuracy, with no restrictions; and the assessment of the risk of bias of the included studies. These characteristics make this review unique for this topic. However, in our systematic review, we decided to exclude studies in which a wearable device was not positioned on a wrist. Some devices can be positioned both on the wrist and other sites (torso, hip, ankle, arm, or brassiere), and the acceptability and accuracy can vary for the same device depending on where it is positioned, increasing heterogeneity [77,78]. Therefore, our results cannot be generalized to the acceptability and accuracy of devices worn on sites other than wrists. Acceptability is defined and measured in many different ways in the literature about wearing devices and about information technology in general [79]. These definitions are often broad and nonspecific, with published literature suggesting that acceptability research should become more robust [80]. For the purpose of our paper, acceptability was operationalized using proxies such as wearing time or data availability. However, other definitions have proposed that acceptability is related more to the extent to which individuals receiving a health care intervention find it appropriate based on cognitive and emotional responses to the intervention [80]. It is important to recognize that acceptability may be more of a holistic and subjective construct rather than an objective one, and thus wear time or data availability may not do full justice to acceptability. Although these metrics have the advantage of being relatively easy to obtain and reproduce, allowing for quantitative comparisons, they are only proxies for acceptability, which is a more nuanced concept. For example, one might wonder if wearing time is low because a person only wears the device a few hours each day or only on weekends or if they completely stopped wearing it after some time. Moreover, wearing time is more likely to offer valuable information in studies with a long follow-up, whereas 2 out of 3 studies reporting on this outcome had a follow-up of <1 week. Because of the presence of important heterogeneity among studies, we were not able to perform a meta-analysis.

Regardless, the comprehensive reporting in this review will allow researchers to assess the available evidence and inform future studies, either to further assess the accuracy of wearable devices or to inform the choice of one device over another to use in interventional studies. To facilitate these choices, we have provided to readers the database with the results of the individual included studies and we did our best to offer a synthesis of the 3 outcomes reported most frequently (step counts, heart rate, and EE).

### Future Research

Further high-quality studies are needed to determine the accuracy and acceptability of wearable devices for measuring physical activity. Given the number of devices available (72 included in this review), it is unlikely that a single study will be able to answer this question. This makes it particularly important to standardize some aspects of these studies, to reduce the heterogeneity among them, and allow for meta-syntheses of the results with comparisons across studies, devices, and outcomes. If the heterogeneity was acceptable, a network meta-analysis would also allow researchers to make indirect comparisons. The main sources of heterogeneity that could be controlled are the setting of the study, the population, the reference standard used, and the outcome definition and measure. A first step in this direction would be putting together a task force of experts to issue guidelines on how to report these experiments, similar to guidelines for the EQUATOR network. A second step would be to issue recommendations on this aspect, starting with accepted reference standards against which devices should be tested for each outcome, the conditions in which the experiment should be conducted, and the way in which the outcomes should be measured and analyzed. Regarding the reference standards, some of these are more accurate than others. Our approach was to take accuracy to mean criterion and convergent validity in this review, but once there is consensus on the acceptable reference standard, other comparisons should not be included in a meta-synthesis. Regarding the method to report on the accuracy of continuous variables (more common in this field), this is the order of priority that we suggest: MAPE, mean percentage error, mean difference, Bland–Altman mean bias, and measure of correlation as the least preferred. This is because the percentage error gives the reader a better understanding of the importance of the error (a mean error of 50 steps is much more relevant if the total step count was 100 than if it was 10,000). We preferred the MAPE over the mean absolute error because when the absolute value is not used, there is a risk of negative and positive errors balancing each other, with the risk of overestimating the accuracy. We prefer the mean difference over the Bland–Altman mean bias because in an accuracy study, the reference standard is supposed to be more accurate than the index test, and therefore the latter should be tested against the former, not against their mean. In the case of the acceptability outcome, consensus should be reached also on how to define and measure it. For example, defining a minimum set of outcomes to be reported might help in this context. This might include reporting the percentage of abandonment over time. Furthermore, as new devices become available, their acceptability and accuracy should also be tested because they could differ from the acceptability and accuracy of other devices, even those produced by the same company. Regarding the choice of the device to use in interventional studies, for example, in studies that aim at increasing physical activity in a certain population, there is no one-device-fits-all answer. This choice should be based on the available data on acceptability and accuracy and be tailored to the outcome to measure. In a study with step count as the main outcome, the Fitbit Charge and Fitbit Charge HR might be appropriate choices. The Apple Watch might be preferred if the main outcome is heart rate. Active time was most often measured through time spent in MVPA, and the Fitbit Flex is the only device that was used in 3 studies, showing good results in 2 of these. Regarding EE, we do not feel comfortable suggesting the use of any device based on the current evidence because the accuracy was poor across devices. The decision should probably be driven by the other outcomes used. Broader recommendations should be issued in the form of guidelines from a panel of experts using this systematic review as a knowledge base.

### Conclusions

We reported on the acceptability and accuracy of 72 wrist-wearable devices for measuring physical activity produced by 29 companies. The Fitbit Charge and Fitbit Charge HR were consistently shown to have a good accuracy for step counts and the Apple Watch for measuring heart rate. None of the tested devices proved to be accurate in measuring EE. Efforts should be made to reduce the heterogeneity among studies.

## Appendix

Multimedia Appendix 1 Database of result characteristics of the studies reporting on accuracy and acceptability.

Multimedia Appendix 2 Guide for accessing database in Multimedia Appendix 1.

Multimedia Appendix 3 Result characteristics of the studies reporting on accuracy.

## Abbreviations

EE: energy expenditure
MAPE: mean absolute percentage error
MVPA: moderate to vigorous-intensity physical activity
