# Supplementary material for: Accuracy and Acceptability of Wrist-Wearable Activity-Tracking Devices: Systematic Review of the Literature
Source: J Med Internet Res. 2022 Jan 21;24(1):e30791. doi: 10.2196/30791 (PMC8817215; doi:10.2196/30791)

Supplementary material:

Outcome definitions:

Mean absolute percentage error (MAPE)

$$Mean\ absolute\ percentage\ error\ (MAPE) = \frac{1}{n}\sum_{t=1}^{n}\left|\frac{A_t - F_t}{A_t}\right|$$

n = number of times the summation iteration happens
A_t = actual value (measured with the reference standard)
F_t = Forecast value (measured with the index test)

Mean percentage error

$$Mean\ percentage\ error = \frac{1}{n}\sum_{t=1}^{n}\frac{A_t - F_t}{A_t}$$

n = number of times the summation iteration happens
A_t = actual value (measured with the reference standard)
F_t = Forecast value (measured with the index test)

Mean difference

$$Mean\ difference = \frac{1}{n}\sum_{t=1}^{n} A_t - F_t$$

n = number of times the summation iteration happens
A_t = actual value (measured with the reference standard)
F_t = Forecast value (measured with the index test)

Mean bias (Bland-Altman)

$$Mean\ bias\ (Bland - Altman) = \frac{1}{n}\sum_{t=1}^{n}(F_t - \frac{(A_t + F_t)}{2})$$

n = number of times the summation iteration happens
A_t = actual value (measured with the reference standard)
F_t = Forecast value (measured with the index test)

Supplementary Table 1: characteristics and results of all the studies included in the review (including the risk of bias assessment for each outcome).

The table was added as an excel file to the supplementary material. The reader can use the filter function at their preference, for example to isolate the objective (accuracy versus acceptability) the outcome within the objective (step count, energy expenditure, …), the device used as the index test, and the reference standard. An example is shown in **Supplementary figures 1 and 2**.

Supplementary Figure 1: accessing the filter function

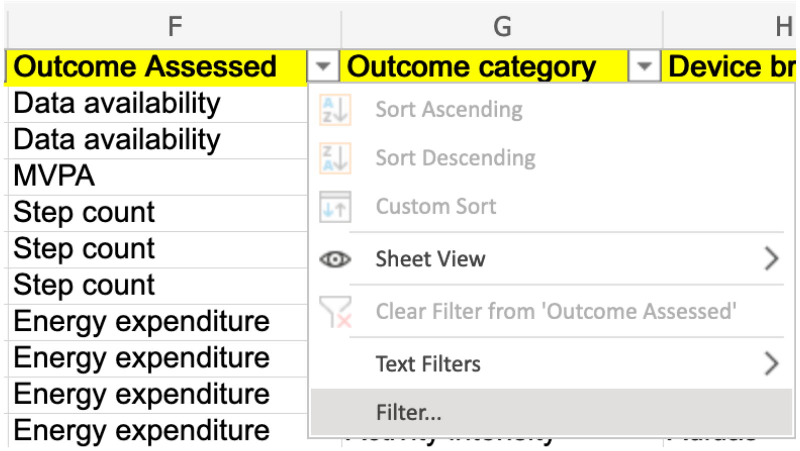

Supplementary Figure 2: filters available for the variable "Outcome Assessed"

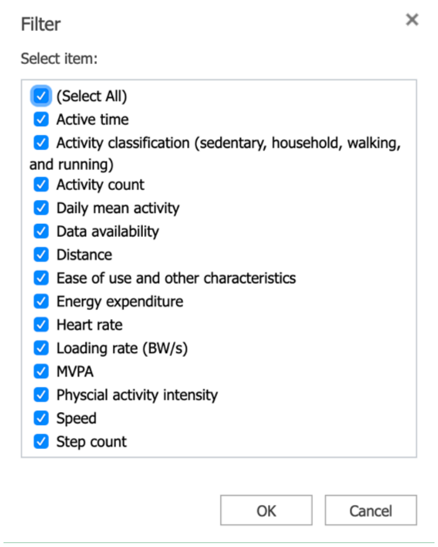

Supplement: Multimedia Appendix 2 [file jmir_v24i1e30791_app2.pdf]
