# Supplementary material for: Accuracy and Acceptability of Wrist-Wearable Activity-Tracking Devices: Systematic Review of the Literature
Source: J Med Internet Res. 2022 Jan 21;24(1):e30791. doi: 10.2196/30791 (PMC8817215; doi:10.2196/30791)
Supplement: Multimedia Appendix 3 [file jmir_v24i1e30791_app3.docx]

| **Author, year** | **Device brand** | **Device model** | **Reference standard** | **Results** | **Scale of Measure** |
| --- | --- | --- | --- | --- | --- |
| **Outcome: Active time** | | | | | |
| Hernandez-Vicente, 2016 | Polar | V800 | ActiTrainer | Mean (SD) bias (Bland-Altman) 32.0 (52.0) min, with mean (SD) 303.95 (93.29) min measured with the reference standard | Activity over 7-days under everyday conditions |
| **Outcome: Activity classification (sedentary, household, walking, and running)** | | | | | |
| Zhang, 2012 | GENE | Activ | Probably direct observation | For different machine learning algorithms (Logistic Regression, Decision Tree, Support Vector Machine, and Bayesian Network) the Incorrect classification rate ranged from 2.71 to 4.44% | 10-12 semi structured activities in lab or outdoor environment while wearing device |
| **Outcome: Activity count** | | | | | |
| Gironda, 2007 | Actiwatch | Score | VICON Motion Analysis System | Pearson’s correlation coefficient 0.67-0.88 | Performance on two 15-minute trials of exercise activity prescribed for back-pain rehab. |
| Lawinger, 2015 | ActiGraph | GT3X+ | Manual count (video recording) | Correlation r .93, P < .001 “every 4000-vector-magnitude physical activity counts equal 27 arm motions”. This 4000 was not pre-specified | Performance on 3 series of tasks: activities of daily living, rehab exercises and passive shoulder range at 5 specified velocities in one lab session. |
| Bruder, 2018 | ActivPAL | ActivPAL | 10-camera 3-D Motion analysis system (Vicon-MX3) | Mean difference -40.9 to 30.4 for different activities (95% CI reported) | Performance on two upper limb activities on week apart |
| **Outcome: Daily mean activity** | | | | | |
| Scott, 2017 | GENE | Activ | ActiGraph GT3X+ | Pearson's r 0.88 (95% CI = 0.82–0.93; p = <0.001) | Activity over 7-days under everyday conditions |
| **Outcome: Distance** | | | | | |
| Gaz, 2018 | Fitbit | Charge HR | Measured distance | Mean (SD) difference 0.028 (0.045) to 0.152 (0.124) m | Performance on a free walking or treadmill walking condition. Treadmill walking had pre-determined speeds. |
| Gaz, 2018 | Apple | Watch, series not NA | Measured distance | Mean (SD) difference 0.016 (0.05)to 0.037 (0.108) |  |
| Gaz, 2018 | Garmin | Vivofit 2 | Measured distance | Mean (SD) difference 0.016 (0.028) to 0.107 (0.066) |  |
| Gaz, 2018 | Jawbone | UP2 | Measured distance | Mean (SD) difference 0.008 (0.049) to 0.086 (0.059) |  |
| Huang, 2016 | Jawbone | Up24 | Measured distance | Mean (SD) percentage error 5.2 (9.8) during flat ground walking (400 m) | Performance on slow, moderate, and fast walking speeds on treadmill  Performance on slow, moderate, and fast walking speeds on treadmill |
| Huang, 2016 | Garmin | Vivofit | Measured distance | Mean (SD) percentage error 5.1 (11.4) during flat ground walking (400 m) |  |
| Huang, 2016 | Fitbit | Flex | Measured distance | Mean (SD) percentage error -12.8 (15.4)% during flat ground walking (400 m) |  |
| Wahl, 2017 | Beurer | AS80 | Measured distance | MAPE -51.9 to -17.6% | Performance on a treadmill for four 5 minute stages of different velocities, a 5-minute period of intermittent velocity, and a 2.4 km outdoor run  and a 2.4 km outdoor run |
| Wahl, 2017 | Fitbit | Charge HR | Measured distance | MAPE -29.5 to -13.1% |  |
| Wahl, 2017 | Fitbit | Charge | Measured distance | MAPE -29.9 to 16% |  |
| Wahl, 2017 | Garmin | Vivofit | Measured distance | MAPE -25.0 to 23.3% |  |
| Wahl, 2017 | Garmin | Vivosmart | Measured distance | MAPE -8.1 to 53.5% |  |
| Wahl, 2017 | Garmin | Vivoactive | Measured distance | MAPE -6.1 to 51.4% |  |
| Wahl, 2017 | Garmin | Forerunner 920XT | Measured distance | MAPE -3.3 to 26.0% |  |
| Wahl, 2017 | Xaomi | Mi Band | Measured distance | Not Applicable (too many missing data, not analyzed) |  |
| Wahl, 2017 | Withings | Pulse | Measured distance | MAPE 0.7 to 58.3% |  |
| **Outcome: Energy expenditure** | | | | | |
| Stackpool, 2013 | Jawbone | UP | Indirect calorimetry (Portable metabolic analyzer) | Pearson's r 0.20 to 0.87 | First session completed on a treadmill at walking or running speed, selected by the participant. Second session was on elliptical cross-trainer at self-selected speed. Apart of the second session also took place in a gymnasium, where they completed agility ladder drills, basketball throws, and basketball lay-ups. |
| Stackpool, 2013 | Nike | Fuelband | Indirect calorimetry (Portable metabolic analyzer) | Pearson's r 0.08 to 0.72 |  |
| Stackpool, 2013 | Fitbit | Ultra | Indirect calorimetry (Portable metabolic analyzer) | Pearson's r 0.24 to 0.67 |  |
| Stackpool, 2013 | Adidas | MiCoach | Indirect calorimetry (Portable metabolic analyzer) | Pearson's r 0.55 to 0.81 |  |
| Compagnat, 2018 | ActiGraph | GT3X+ | Indirect calorimetry (portable gas analyser, Metamax 3B, Cortex) | Mean percentage difference 3% for walking subjects, 47% for subjects with wheelchair | Performed four tasks: transfers, manual tasks, walking on flat ground and walking up and down stairs. |
| Hargens, 2017 | Fitbit | Charge | ActiGraph GT3x | MAPE 30.6% | Activity over 7-days under everyday conditions |
| Mandigout, 2017 | Actical | Actical | Indirect calorimetry (portable gas analyser, Metamax 3B, Cortex) | Spearman's r –0.19 (p 0.35) if weared on the plegic side, –0.27 (p 0.23) on the non-plegic side | Performance in various everyday tasks (transfer, walking, etc) within a laboratory setting |
| Mandigout, 2017 | ActiGraph | GTX | Indirect calorimetry (portable gas analyser, Metamax 3B, Cortex) | Spearman's r 0.08 (p 0.71) if wore on the plegic side, 0.20 (p 0.34) on the non-plegic side |  |
| Montoye, 2017 | Fitbit | Charge HR | Indirect calorimetry (Parvo metabolic analyzer) | MAPE (SD) 43.7 (3.4) | Performing 14 activities in a laboratory and on a track (lying, sitting, standing, walking various speed and inclines, jogging, and cycling) |
| Price, 2017 | Fitbit | One | Indirect calorimetry using ParvoMedics TrueOne 2400 | Mean (SD) bias 2.91 (4.35) kcals/min | Walking on a treadmill at varying speeds |
| Price, 2017 | Garmin | Vivofit | Indirect calorimetry using ParvoMedics TrueOne 2400 | Mean (SD) bias -1.56 (2.34) kcals/min |  |
| Price, 2017 | Jawbone | UP | Indirect calorimetry using ParvoMedics TrueOne 2400 | Mean (SD) bias 18.57 (30.17) kcals/min |  |
| Roos, 2017 | Suunto | Ambi | Indirect calorimetry | MAPE 21.32 to 41.93% | Aerobic and anaerobic running on a treadmill in a laboratory setting |
| Roos, 2017 | Garmin | Forerunner 920XT | Indirect calorimetry | MAPE 11.54 to 49.30% |  |
| Roos, 2017 | Polar | V800 | Indirect calorimetry | MAPE 10.1 to 39.5% |  |
| Alsubheen, 2016 | Garmin | Vivofit | Indirect Calorimetry (Sable Systems International, Las Vegas NV) | Systematically underestimated by 29.5% during treatdmill walking test, p | Performance on treadmill walking tasks, and office activities within a laboratory session, completed in separate sessions on different days |
| Boeselt, 2016 | Polar | A300 | BodyMedia SenseWear | Pearson's r 0.74 (p < 0.01) | Performance in everyday conditions |
| Choi, 2010 | ActiGraph | GT1M | Room calorimeter | Mean (SD) percentage difference: 0.5 (8.0)% | Monitored through a 24-h stay in a laboratory setting. Stay included light activities, eating, sleeping, and participants were encouraged to complete normal day activities during downtime. |
| Chowdhury, 2017 | Microsoft | Band | CamNtech Actiheart | MAPE (SD) 34 (10)% | Performance against criterion measurements in both controlled laboratory conditions (simulated activities of daily living and structured exercise) and over a 24-hour period in free-living conditions. |
| Chowdhury, 2017 | Apple | Watch, series not NA | CamNtech Actiheart | MAPE (SD) 15 (10)% |  |
| Chowdhury, 2017 | Jawbone | Up24 | CamNtech Actiheart | MAPE (SD) 30 (11)% |  |
| Chowdhury, 2017 | Fitbit | Charge | CamNtech Actiheart | MAPE (SD) 16 (8)% |  |
| Chowdhury, 2017 | Microsoft | Band | Indirect calorimetry (portable gas analyser, COSMED K4b2) | MAPE (SD) 40 (16)% |  |
| Chowdhury, 2017 | Apple | Watch, series not NA | Indirect calorimetry (portable gas analyser, COSMED K4b2) | MAPE (SD) 27 (19)% |  |
| Chowdhury, 2017 | Jawbone | Up24 | Indirect calorimetry (portable gas analyser, COSMED K4b2) | MAPE (SD) 36 (14)% |  |
| Chowdhury, 2017 | Fitbit | Charge | Indirect calorimetry (portable gas analyser, COSMED K4b2) | MAPE (SD) 36 (22)% |  |
| Dondzila, 2018 | Fitbit | Charge HR | MET values of treadmill intensities | MAPE -8.4 to 89.2% | Performance on four-five minute stage treadmill tasks in a laboratory session and later in free-living conditions for one day. |
| Dondzila, 2018 | Miio | FUSE | MET values of treadmill intensities | MAPE 0 to 44.9% |  |
| Durkalec-Michalski, 2013 | ActiGraph | GT1M | Indirect Calorimetery | Overestimated EE at moderate intensity by 60% and underestimated EE by 40% at vigorous intensity. 86% accurate in measuring EE at light intensity in relation to the values measured by indirect calorimetry. | Performance on leisure and exercise activities at various intensities in laboratory and free-living conditions |
| Ferguson, 2015 | Misfit | Shine | BodyMedia SenseWear | Mean absolute difference 468, mean (SD) measured with the reference standard = 3005 (569) | Activity under free-living conditions over 48 hours |
| Ferguson, 2015 | Jawbone | UP | BodyMedia SenseWear | Mean absolute difference 866, mean (SD) with the reference standard = 3005 (569) |  |
| Hernandez-Vicente, 2016 | Polar | V800 | Actigraph ActiTrainer | Mean (SD) bias (Bland-Altman) 957.5 (679.9) kcal, with mean (SD) 1,456.48 (731.40) kcals measured with the reference standard | Activity under free-living conditions over 7 days |
| Lemmens, 2018 | Phillips | Optical Heart Rate monitor | Indirect calorimetry (portable gas analyser, COSMED K4b2) | Mean percentage error -2.6% | Performance on paced and self-paced exercise activities as well as household activities under laboratory conditions |
| Sirard - Phase 2 (Lab), 2017 | Movband | Movband | Indirect calorimetry system (Oxycon Mobile, Carefusion, Inc.) | Spearman's r 0.61 | Performance on structured activities (sitting, self-paced walking, catch, tag, jogging) within a laboratory condition over 2 days |
| Sirard - Phase 2 (Lab), 2017 | Sqord | Sqord | Indirect calorimetry system (Oxycon Mobile, Carefusion, Inc.) | Spearman's r 0.87 |  |
| Wallen, 2016 | Apple | Watch, series not NA | Indirect calorimetry (MetaMax 3B, Cortex, Germany) | Mean (SD) bias (Bland-Altman) -123.1 (55.6) kcal, with index test mean (SD) = 285.7 (50.2) | Completed ~1-hr protocols involving supine and seated rest, walking and running on a treadmill and cycling on an ergometer in a laboratory condition |
| Wallen, 2016 | Fitbit | Charge | Indirect calorimetry | Mean (SD) bias (equation reported, since Bland-Altman parameters were systematically biased) 0.61*mean–224.6 (59.1) kcal, with index test mean (SD) = 236.8 (77.0) |  |
| Wallen, 2016 | Samsung | Gear S | Indirect calorimetry | Mean (SD) bias (Bland-Altman) -26.1 (24.2) kcal, with index test mean (SD) = 261.4 (47.5) |  |
| Wallen, 2016 | Miio | Mio alpha | Indirect calorimetry | Mean (SD) bias (equation reported, since Bland-Altman parameters were systematically biased) 0.91*mean -318.77 (84.8) kcal, with index test mean (SD) = 236.8 (77.0) |  |
| Woodman, 2017 | Garmin | Vivofit | Indirect calorimetry (Oxycon Mobile, Carefusion, Inc.) | MAPE (SD) 44.6 (~8) | Completed 11 activities ranging from sedentary behaviors to vigorous intensities in a laboratory condition over one day |
| Woodman, 2017 | Withings | Pulse | Indirect calorimetry (Oxycon Mobile, Carefusion, Inc.) | MAPE (SD) 63.7 (~4.5) |  |
| Woodman, 2017 | Basis | Peak | Indirect calorimetry (Oxycon Mobile, Carefusion, Inc.) | MAPE (SD) 27.2 (~20) |  |
| Imboden, 2018 | Fitbit | Flex | Indirect calorimetry | Mean percentage bias = -13% | Participated in an 80-minute protocol of exercises in a laboratory condition |
| Imboden, 2018 | Jawbone | Up24 | Indirect calorimetry | Mean percentage bias = -26% |  |
| Wahl, 2017 | Polar | Loop | Indirect calorimetry with portable gas analyzer Metamax 3B (Metamax 3B, CORTEX Biophysik GmbH, Leipzig, Germany) | MAPE 5.6 to 56.4% | Performed a running protocol consisting of four 5 min stages of different constant velocities (4.3; 7.2; 10.1; 13.0 km·h−1), a 5 min period of intermittent velocity, and a 2.4 km outdoor run (10.1 km·h−1). |
| Wahl, 2017 | Beurer | AS80 | Indirect calorimetry with portable gas analyzer Metamax 3B (Metamax 3B, CORTEX Biophysik GmbH, Leipzig, Germany) | MAPE -48.4 to 17% |  |
| Wahl, 2017 | Fitbit | Charge HR | Indirect calorimetry with portable gas analyzer Metamax 3B (Metamax 3B, CORTEX Biophysik GmbH, Leipzig, Germany) | MAPE -12.0 to 83.3% |  |
| Wahl, 2017 | Fitbit | Charge | Indirect calorimetry with portable gas analyzer Metamax 3B (Metamax 3B, CORTEX Biophysik GmbH, Leipzig, Germany) | MAPE -4.5 to 75.0% |  |
| Wahl, 2017 | Bodymedia | Sensewear | Indirect calorimetry with portable gas analyzer Metamax 3B (Metamax 3B, CORTEX Biophysik GmbH, Leipzig, Germany) | MAPE -25.3 to -1.4% |  |
| Wahl, 2017 | Garmin | Vivofit | Indirect calorimetry with portable gas analyzer Metamax 3B (Metamax 3B, CORTEX Biophysik GmbH, Leipzig, Germany) | MAPE -21.3 to 18.7% |  |
| Wahl, 2017 | Garmin | Vivosmart | Indirect calorimetry with portable gas analyzer Metamax 3B (Metamax 3B, CORTEX Biophysik GmbH, Leipzig, Germany) | MAPE -1.5 to -35.8% |  |
| Wahl, 2017 | Garmin | Vivoactive | Indirect calorimetry with portable gas analyzer Metamax 3B (Metamax 3B, CORTEX Biophysik GmbH, Leipzig, Germany) | MAPE -4.5 to 36.8% |  |
| Wahl, 2017 | Garmin | Forerunner 920XT | Indirect calorimetry with portable gas analyzer Metamax 3B (Metamax 3B, CORTEX Biophysik GmbH, Leipzig, Germany) | MAPE -26.6 to -9.2% |  |
| Wahl, 2017 | Xaomi | Mi Band | Indirect calorimetry with portable gas analyzer Metamax 3B (Metamax 3B, CORTEX Biophysik GmbH, Leipzig, Germany) | Not applicable (too many missing data, not analyzed) |  |
| Wahl, 2017 | Withings | Pulse | Indirect calorimetry with portable gas analyzer Metamax 3B (Metamax 3B, CORTEX Biophysik GmbH, Leipzig, Germany) | MAPE -38.9 to -16.9% |  |
| Dooley, 2017 | Apple | Watch, series not NA | Indirect calorimetry with Parvo Medics TrueOne 2400 (Parvo Medics Inc, Sandy, UT, USA) | MAPE (SD) 16.54 (~13) to 210.84 (~96)% | Participants completed a 10-minute seated baseline assessment; separate 4-minute stages of light-, moderate-, and vigorous-intensity treadmill exercises; and a 10-minute seated recovery period in a laboratory setting |
| Dooley, 2017 | Fitbit | Charge HR | Indirect calorimetry with Parvo Medics TrueOne 2400 (Parvo Medics Inc, Sandy, UT, USA) | MAPE (SD) 16.85 (~14) to 84.98 (~46)% |  |
| Dooley, 2017 | Garmin | Forerunner 225 | Indirect calorimetry with Parvo Medics TrueOne 2400 (Parvo Medics Inc, Sandy, UT, USA) | MAPE (SD) 30.77 (~26) to 155.05 (~164)% |  |
| **Outcome: Heart rate** | | | | | |
| Jo, 2016 | Basis | Peak K | Standard 12- lead electrocardiograph system (Cosmed C12x; Concord,  CA, USA) | Mean(SD) bias (Bland-Altman) -3 (11) bpm | Each participant completed an initial rest period of 15 minutes followed by 5-minute periods of each of the following activities: 60W and 120W cycling, walking, jogging, running, resisted arm raises, resisted lunges, and isometric plank. In between each exercise task was a 5-minute rest period. |
| Jo, 2016 | Fitbit | Charge | Standard 12- lead electrocardiograph system (Cosmed C12x; Concord,  CA, USA) | Mean(SD) bias (Bland-Altman) -9 (17) bpm |  |
| Montoye, 2017 | Fitbit | Charge HR | Nonin PureSAT Pulse Oximeter | MAPE (SD) 6.6 (0.6) | Performing 14 activities in a laboratory and on a track (lying, sitting, standing, walking various speed and inclines, jogging, and cycling) |
| Dondzila, 2018 | Fitbit | Charge HR | Polar heart rate monitor | Trend to report lower mean heart rate values at running speeds of 134.1 m·min-1 and 160.9 m·min-1, compared to the Polar. | Performance on four-five minute stage treadmill tasks in a laboratory session and later in free-living conditions for one day. |
| Dondzila, 2018 | Miio | FUSE | Polar heart rate monitor | Mean heart rate values within 1.1 beats·min-1 of the Polar. |  |
| Gillinov, 2017 | Garmin | Forerunner 235 | ECG leads, polar H7 chest strap monitor, scosche rhythm on forearm | MAPE (SD) 4.6 (7.7) to 13.7 (16.8)% | Completed exercise protocols on a treadmill, a stationary bicycle, and an elliptical trainer (Tarm movement) in a laboratory setting over one day. |
| Gillinov, 2017 | TomTom | Spark | ECG leads, polar H7 chest strap monitor, scosche rhythm on forearm | MAPE (SD) 4.5 (5.3) to 6.7 (9.6)% |  |
| Gillinov, 2017 | Apple | Watch, series not NA | ECG leads, polar H7 chest strap monitor, | MAPE (SD) 3.2 (4.9) to 6.5 (10.8)% |  |
| Gillinov, 2017 | Fitbit | Blaze | ECG leads, polar H7 chest strap monitor, scosche rhythm on forearm | MAPE (SD) 5.6 (6.4) to 15.9 (18.2)% |  |
| Powierza, 2017 | Fitbit | Charge | Electrocardiogram | Mean(SD) bias (Bland-Altman) -6.04 (10.40) bpm | Completed the Buffalo Concussion Treadmill Test in a laboratory setting over one day. |
| Støve, 2019 | Garmin | Forerunner | Polar device | Mean difference (SD) 1 (2.3) to 17 (13.36) bpm, with mean (SD) frequence ranging from 59.5 (10.8) to 165.8 (16.4) | Performance during rest and three exercise conditions at submaximal level including cycling, treadmill, walking, running and rapid arm movement in a laboratory setting |
| Thomson, 2019 | Apple | Watch, series not NA | ECG | Mean percentage error 2.4 to 5.1% | Measured over performance in different intensity levels of activity, from very light to very rigorous, in a laboratory session over 1 day. |
| Thomson, 2019 | Fitbit | Charge HR 2 | Electrocardiogram | Mean percentage error 3.9 to 13.5% |  |
| Wallen, 2016 | Apple | Watch, series not NA | ECG | Mean (SD) bias (Bland-Altman) -1.3 (4.4) bpm, with index test mean (SD) = 102.0 (14.4) | Completed ~1-hr protocols involving supine and seated rest, walking and running on a treadmill and cycling on an ergometer in a laboratory condition |
| Wallen, 2016 | Fitbit | Charge | Electrocardiogram and indirect calorimetry | Mean (SD) bias (Bland-Altman) -9.3 (8.5) bpm, with index test mean (SD) = 102.0 (14.5) |  |
| Wallen, 2016 | Samsung | Gear S | Electrocardiogram and indirect calorimetry | Mean (SD) bias (Bland-Altman) -7.1 (10.3) bpm, with index test mean (SD) = 100.5 (14.6) |  |
| Wallen, 2016 | Miio | Mio alpha | Electrocardiogram and indirect calorimetry | Mean (SD) bias (Bland-Altman) -4.3 (7.2) bpm, with index test mean (SD) = 102.0 (14.4) |  |
| Dooley, 2017 | Apple | Watch, series not NA | ActiGraph GT3X+, Polar Heart Rate Monitor, Pravo Medica TrueOne 2400 | MAPE (SD) 1.4 (~1) to 6.7 (~11)% | Participants completed a 10-minute seated baseline assessment; separate 4-minute stages of light-, moderate-, and vigorous-intensity treadmill exercises; and a 10-minute seated recovery period in a laboratory setting |
| Dooley, 2017 | Fitbit | Charge HR | ActiGraph GT3X+, Polar Heart Rate Monitor, Pravo Medica TrueOne 2400 | MAPE (SD) 2.4 (~1.5) to 17.0 (~20.0) |  |
| Dooley, 2017 | Garmin | Forerunner 225 | ActiGraph GT3X+, Polar Heart Rate Monitor, Pravo Medica TrueOne 2400 | MAPE (SD) ranging from 7.8 (~17) to 24.38 (~26) |  |
| Stiles, 2013 | GENE | Activ | Advanced Mechanical Technology Inc. force plate | Sensitivity 97.6%, specificity 75.0%, overall agreement 85.6%, using a cut-off point of 3.125 g | Performed walking (slow, fast, and with bag), floor sweeping, running (slow and fast), jumping (low, G5 cm; high, 95 cm), and box drop (20 cm) in a laboratory session. |
| Stiles, 2013 | ActiGraph | GT3X+ | Advanced Mechanical Technology Inc. force plate | Sensitivity 90.5%, specificity 81.3%, overall agreement 85.6 using a pre-specified cut-off cut-off point 2.840 g |  |
| **Outcome: MVPA** |  |  |  |  |  |
| Semanik, 2020 | Fitbit | Flex | ActiGraph GT3X | Mean (SD) difference 18.5 (11.3), with mean (SD) 239.5 (86.2) min/day measured with the reference standard | Activity over 7-days under everyday conditions |
| Hargens, 2017 | Fitbit | Charge | ActiGraph GT3x | MAPE 46.3% | Activity over 7-days under everyday conditions |
| Scott, 2017 | GENE | Activ | ActiGraph GT3X+ | Pearson's r 0.84 (95% CI = 0.77–0.89; p <0.001) | Activity over 7-days under everyday conditions |
| Boeselt, 2016 | Polar | A300 | Bodymedia-SenseWear (SWA) device | Pearson's r -0.25 (p <0.01) | Performance in everyday conditions |
| Ferguson, 2015 | Misfit | Shine | Actigraph GT3X+ | Mean absolute difference (MAD) = 15.2, mean (SD) with the reference standard = 58.5 (37.6) | Activity under free-living conditions over 48 hours |
| Ferguson, 2015 | Jawbone | UP | Actigraph GT3X+ | Mean absolute difference (MAD) = 18.0, mean (SD) with the reference standard = 58.5 (37.6) |  |
| Redenius, 2019 | Fitbit | Flex | Actigraph GT3X+ | MAPE (SD) 6.7 (5.7) to 74.3 (12.8)% | Activity over 7-days under everyday conditions |
| Reid, 2017 | Fitbit | Flex | Actigraph GT3X+ | Mean (SD) bias (Bland-Altman) -57.5 (46.4) min/day, with mean 64.6 min/day measured with the reference standard | Activity over 7-days under everyday conditions |
| Sirard - Phase 3 (Field), 2017 | Movband | Movband | ActiGraph GT3X+ | Spearman's r 0.76 | Activity over 4-days under everyday conditions |
| Sirard - Phase 3 (Field), 2017 | Sqord | Sqord | ActiGraph GT3X+ | Spearman's r 0.86 |  |
| St-Laurent, 2018 | Fitbit | Flex | Actigraph GT3x | Mean (SD) bias (Bland-Altman) 2.4 ± 6.6 (p = 0.21) min/day, with mean (SD) 9.9 (7.5) min/day measured with the reference standard | Activity over 7-days under everyday conditions |
| Alharbi, 2016 | Fitbit | Flex | Actigraph | Mean percentage error 10% | Activity over 4-days under everyday conditions |
| Imboden, 2018 | Fitbit | Flex | ActiGraph GT3X+ | Mean percentage error -65% | Participated in an 80-minute protocol of exercises in a laboratory condition |
| Imboden, 2018 | Jawbone | Up24 | ActiGraph GT3X+ | Mean percentage error -35% |  |
| **Outcome: Physcial activity intensity** | | | | | |
| Bulathsinghala, 2014 | ActiGraph | GT3X+ | ActiGraph GT3X+ on the waist | Physical activity intensity above the threshold was present in16% of the recorded minutes. Mean Vector Magnitude Unit (VMU - movement in three planes) from the wrist device above the 3000 threshold were 4953 (95% confidence interval (CI), 4850 to 5055), while corresponding VMU from the waist device were 951 (95% CI, 916 to 986). Using a proprietary software equation developed for the waist location, activity intensity above this threshold corresponded to 1.66 metabolic units (METs) (95% CI, 1.55 to 1.77). | Activity over 24 hours under everyday conditions |
| **Outcome: Speed** | | | | | |
| Cohen, 2010 | ActiGraph | Mini MotionLogger | Actual speed (distance/time) | Mean difference 0.97 mph (95% CI, 0.73 - 2.67) | Completed a standardized sequence of activities that comprised sitting, standing, and walking in laboratory setting |
| **Outcome: Step count** | | | | | |
| Stackpool, 2013 | Jawbone | UP | Manual count | Pearson's r 0.34 to 0.99 | First session completed on a treadmill at walking or running speed, selected by the participant. Second session was on elliptical cross-trainer at self-selected speed. Apart of the second session also took place in a gymnasium, where they completed agility ladder drills, basketball throws, and basketball lay-ups. |
| Stackpool, 2013 | Nike | Fuelband | Manual count | Pearson's r 0.17 to 0.98 |  |
| Stackpool, 2013 | Fitbit | Ultra | Manual count | Pearson's r 0.44 to 0.99 |  |
| An, 2017 | Fitbit | Flex | Manual count (Tally Counter) for lab setting, New Lifestyle (NL-1000 Series) pedometer for field setting | MAPE 4.7 to 21.9% in lab, 18.1% on field | Walking/jogging on a treadmill, walking over-ground on an indoor track, and a 24-hour free-living condition |
| An, 2017 | Garmin | Vivofit | Manual count (Tally Counter) for lab setting, New Lifestyle (NL-1000 Series) pedometer for field setting | MAPE 2.4 to 16.5% in lab, 17.8% on field |  |
| An, 2017 | Polar | Loop | Manual count (Tally Counter) for lab setting, New Lifestyle (NL-1000 Series) pedometer for field setting | MAPE 9.9 to 23.8% in lab, 26.9% on field |  |
| An, 2017 | Basis | B1 Band | Manual count (Tally Counter) for lab setting, New Lifestyle (NL-1000 Series) pedometer for field setting | MAPE 3.1 to 9.0% in lab, 18.4% on field |  |
| An, 2017 | Misfit | Shine | Manual count (Tally Counter) for lab setting, New Lifestyle (NL-1000 Series) pedometer for field setting | MAPE 6.3 to 19.3% in lab, 23.3% on field |  |
| An, 2017 | Jawbone | UP24 | Manual count (Tally Counter) for lab setting, New Lifestyle (NL-1000 Series) pedometer for field setting | MAPE 2.9 to 7.0% in lab, 27.9% on field |  |
| An, 2017 | Nike | FuelBand SE | Manual count (Tally Counter) for lab setting, New Lifestyle (NL-1000 Series) pedometer for field setting | MAPE 10.2 to 45.0% in lab, 16.0% on field |  |
| Gaz, 2018 | Fitbit | Charge HR | Manual count (Tally counter) | Mean (SD) difference 21.81 (67.08) to 195.06 (207.94) steps. Max distance 1.6 km. | Performance on a free walking or treadmill walking condition. Treadmill walking had pre-determined speeds |
| Gaz, 2018 | Apple | Watch, series not NA | Manual count (Tally counter) | Mean (SD) difference 7.56 (29.61) to 39.44 (151.81) steps. Max distance 1.6 km. |  |
| Gaz, 2018 | Garmin | Vivofit 2 | Manual count (Tally counter) | Mean (SD) difference 5.09 (8.38) to 98.06 (137.49) steps. Max distance 1.6 km. |  |
| Gaz, 2018 | Jawbone | UP2 | Manual count (Tally counter) | Mean (SD) difference 16.19 (29.14) to 64 (66.32) steps. Max distance 1.6 km. |  |
| Hargens, 2017 | Fitbit | Charge | ActiGraph GT3x | MAPE 20.7% | Activity over 7-days under everyday conditions |
| Jones, 2018 | Fitbit | Flex | Manual count (video) | MAPE 0-4% | Completed treadmill protocol at jogging and running speeds (8km/h-16km/h) in laboratory settings |
| Lauritzen, 2013 | Fitbit | Ultra | Manual count (video) | MAPE (SD) 99.6 (0.8)% | Walking procedure of a straight path over 20m in a laboratory setting |
| Magistro, 2018 | ADAMO | Care Watch | Manual count (Tally counter) | MAPE (SD) -17.70 (20.77) % to -1.10 (2.30) % | Performance on randomly ordered tasks: walking slow, normal and fast self-paced speeds, and up/down stairs in a laboratory setting |
| Montoye, 2017 | Fitbit | Charge HR | Omron HJ 323u Pedometer (Omron Corp., Osaka, Japan) | MAPE (SD) 9.7% (1.2) | Performing 14 activities in a laboratory and on a track (lying, sitting, standing, walking various speed and inclines, jogging, and cycling) |
| Alsubheen, 2016 | Garmin | Vivofit | Kinematics analysis (video camera Sony-HDR-FX1 12X HD, Mini DV Camcorder) | Vivofit systematically underestimated step count only at 0% treadmill inclination | Performance on treadmill walking tasks, and office activities within a laboratory session, completed in separate sessions on different days |
| Falgoust, 2018 | Fitbit | Charge HR | Manual count (Tally counter) | Mean difference - 60.8 steps (p 0.01) | Performance on track laps in laboratory settings |
| Falgoust, 2018 | Fitbit | Surge | Manual count (Tally counter) | Mean difference -86.0 steps (p 0.004) |  |
| Falgoust, 2018 | Garmin | Vivoactive HR | Manual count (Tally counter) | Mean difference -19.7 steps (p 0.03) |  |
| Blondeel, 2018 | Fitbit | Alta | Dynaport Movemonitor (accellerometer) | Mean difference (SD) 773 (829) steps (p=0.009) | Activity over 14-days under everyday conditions |
| Boeselt, 2016 | Polar | A300 | BodyMedia SenseWear | Pearson's r 0.96 (p < 0.01) | Performance in everyday conditions |
| Burton, 2018 | Fitbit | Flex | Manual count (video) by two researchers | Intraclass Correlation (ICC) 0.77 (0.57,0.88) and 0.76 (0.53,0.88) in two 2-minutes walking tests | Two 2-minute walk tests were completed while wearing the fitness trackers. Participants were videoed during each test. Participants were then given one fitness tracker and an accelerometer to wear at home for 14-days. |
